# microRNA-483 ameliorates hypercholesterolemia by inhibiting PCSK9 production

**DOI:** 10.1172/jci.insight.143812

**Published:** 2020-12-03

**Authors:** Jianjie Dong, Ming He, Jie Li, Ariane Pessentheiner, Chen Wang, Jin Zhang, Yameng Sun, Wei-Ting Wang, Yuqing Zhang, Junhui Liu, Shen-Chih Wang, Po-Hsun Huang, Philip L.S.M. Gordts, Zu-Yi Yuan, Sotirios Tsimikas, John Y.J. Shyy

**Affiliations:** 1 Department of Cardiology, First Affiliated Hospital, and; 2 Cardiovascular Research Center, School of Basic Medical Sciences, First Affiliated Hospital, Xi’an Jiaotong University, Xi’an, China.; 3 Division of Cardiology and; 4 Division of Endocrinology and Metabolism, Department of Medicine, University of California, San Diego, La Jolla, California, USA.; 5 Department of Clinical Laboratory, First Affiliated Hospital, Xi’an Jiaotong University, Xi’an, China.; 6 Department of Anesthesiology,; 7 Cardiovascular Research Center,; 8 Department of Critical Care Medicine, Taipei Veterans General Hospital, and; 9 Institute of Clinical Medicine, National Yang-Ming University, Taipei, Taiwan.

**Keywords:** Metabolism, Vascular Biology, Atherosclerosis, Cholesterol, Lipoproteins

## Abstract

Proprotein convertase subtilisin/kexin type 9 (PCSK9) affects cholesterol homeostasis by targeting hepatic LDL receptor (LDLR) for lysosomal degradation. Clinically, PCSK9 inhibitors effectively reduce LDL-cholesterol (LDL-C) levels and the incidence of cardiovascular events. Because microRNAs (miRs) are integral regulators of cholesterol homeostasis, we investigated the involvement of miR-483 in regulating LDL-C metabolism. Using in silico analysis, we predicted that miR-483-5p targets the 3′-UTR of *PCSK9* mRNA. In HepG2 cells, miR-483-5p targeted the *PCSK9* 3′-UTR, leading to decreased PCSK9 protein and mRNA expression, increased LDLR expression, and enhanced LDL-C uptake. In hyperlipidemic mice and humans, serum levels of total cholesterol and LDL-C were inversely correlated with miR-483-5p levels. In mice, hepatic miR-483 overexpression increased LDLR levels by targeting *Pcsk9*, with a significant reduction in plasma total cholesterol and LDL-C levels. Mechanistically, the cholesterol-lowering effect of miR-483-5p was significant in mice receiving AAV8 PCSK9-3′-UTR but not *Ldlr*-knockout mice or mice receiving AAV8 PCSK9-3′-UTR (ΔBS) with the miR-483-5p targeting site deleted. Thus, exogenously administered miR-483 or similarly optimized compounds have potential to ameliorate hypercholesterolemia.

## Abstract 2

MicroRNA-483 targeting 3’ untranslated region of PCSK9 mRNA inhibits hepatic PCSK9 expression, resulting in increased LDLR expression and LDL uptake, which ameliorates hypercholesterolemia and atherosclerosis.

## Introduction

Hypercholesterolemia is a major risk factor for cardiovascular disease. Circulatory levels of low-density lipoprotein cholesterol (LDL-C) are predominantly determined by hepatic clearance of LDL-C via the LDL receptor (LDLR). Proprotein convertase subtilisin/kexin type 9 (PCSK9) plays an important role in cholesterol metabolism by targeting the LDLR (1, 2). PCSK9 is mainly produced by hepatocytes, where its expression is regulated at the transcriptional level by SREBP-2 (encoded by *SREBF2*), a membrane-bound transcription factor that regulates multiple genes involved in cholesterol homeostasis, including *LDLR* (3). Intracellular PCSK9 binds to the nascent LDLR in the trans-Golgi apparatus and renders it for lysosomal degradation (4). Once secreted from hepatocytes, PCSK9 binds to LDLR on the cell surface to form a LDLR–PCSK9 complex. This complex hinders the endocytic recycling of LDLR for lysosomal degradation (5, 6). In concert, these 2 mechanisms effectively reduce LDLR cell surface presentation and LDL-C influx in hepatocytes, thus increasing circulating LDL-C levels.

The importance of PCSK9 in hypercholesterolemia was initially revealed by a genetic analysis of French families with severe autosomal dominant hypercholesterolemia due to gain-of-function mutation of *PCSK9* (7). Then, individuals with loss-of-function mutations (e.g., C679X, R46L) were found to have significantly lower LDL-C levels (8). This central role of PCSK9 in cholesterol homeostasis has led to the development of PCSK9 mAbs (e.g., evolocumab and alirocumab) as well as *PCSK9* siRNA (e.g., inclisiran) (9), which are proposed to be used as monotherapy or combined with statins to reduce LDL-C levels and cardiovascular incidence (10). However, statins are known to elevate the expression of both PCSK9 and LDLR via SREBP-2, thereby attenuating their cholesterol-lowering effect (3, 11). In addition, PCSK9 mAbs are expensive, which questions their cost-effectiveness, and in a few patients, they may induce side effects such as flu-like symptoms. Because of its striking effect on LDLR suppression, hepatic overexpression of *PCSK9* is commonly used to induce hyperlipidemia and accelerate atherosclerosis in rodent models (12).

microRNAs (miRs) are ubiquitously involved in epigenetic regulation of gene expression by targeting the 3′-UTR of the protein-encoded transcripts. Ample evidence demonstrates that miRs are integral regulatory parts of the transcriptome, including genes involved in regulating cholesterol metabolism. For example, miR-96/182/183 target MED1/FBXW7 in hepatic cells, which regulates the transcription of multiple lipid-regulating and lipid synthesis genes (13, 14). miR-27 targets genes involved in lipid deposition, triglyceride synthesis, and lipoprotein uptake in the liver (15). Encoded simultaneously with *SREBF2* and *SREBF1*, miR-33 downregulates cholesterol efflux and HDL biogenesis by targeting ABC transporters, i.e., ABCA1 and ABCG1 (16). Although miRs have been suggested to regulate PCSK9 (17), the full extent of the miRNA biology involvement in regulating PCSK9 and circulatory LDL-C levels remains elusive.

miR-483, containing both miR-483-3p and miR-483-5p (generated from the 3′ and 5′ arms of pre–miR-483), are intronic miRs that are encoded together with their parental gene *IGF2*. The sequences of miR-483-3p/-5p are highly conserved among mammalian species in general (Supplemental Figure 1; supplemental material available online with this article; https://doi.org/10.1172/jci.insight.143812DS1). miR-483 targets the 3′-UTR of connective tissue growth factor (*CTGF*), platelet-derived growth factor, tissue inhibitor of metalloproteinase 2, and *SMAD4*, a key component protein in the TGF-β signaling pathway (18–20). In vascular smooth muscle cells, miR-483 expression is decreased when angiotensin II receptor type 1 is persistently activated (21).

In this study, we uncovered a mechanism by which miR-483-5p robustly targets the 3′-UTR of *PCSK9* mRNA. Experimentally, miR-483-5p directly targeted the 3′-UTR of *PCSK9* mRNA, which in turn increased hepatic LDLR expression. The circulatory levels of miR-483-5p were lower in humans with elevated LDL-C levels, and miR-483 overexpression in hypercholesterolemic mouse models greatly reduced the serum levels of total cholesterol and LDL-C. This mechanism of cholesterol lowering via intracellular *PCSK9* mRNA targeting by miR-483 may have therapeutic efficacy.

## Results

### miRs targeting Pcsk9 alleviated hyperlipidemia and atherosclerosis.

**Figure 1:**
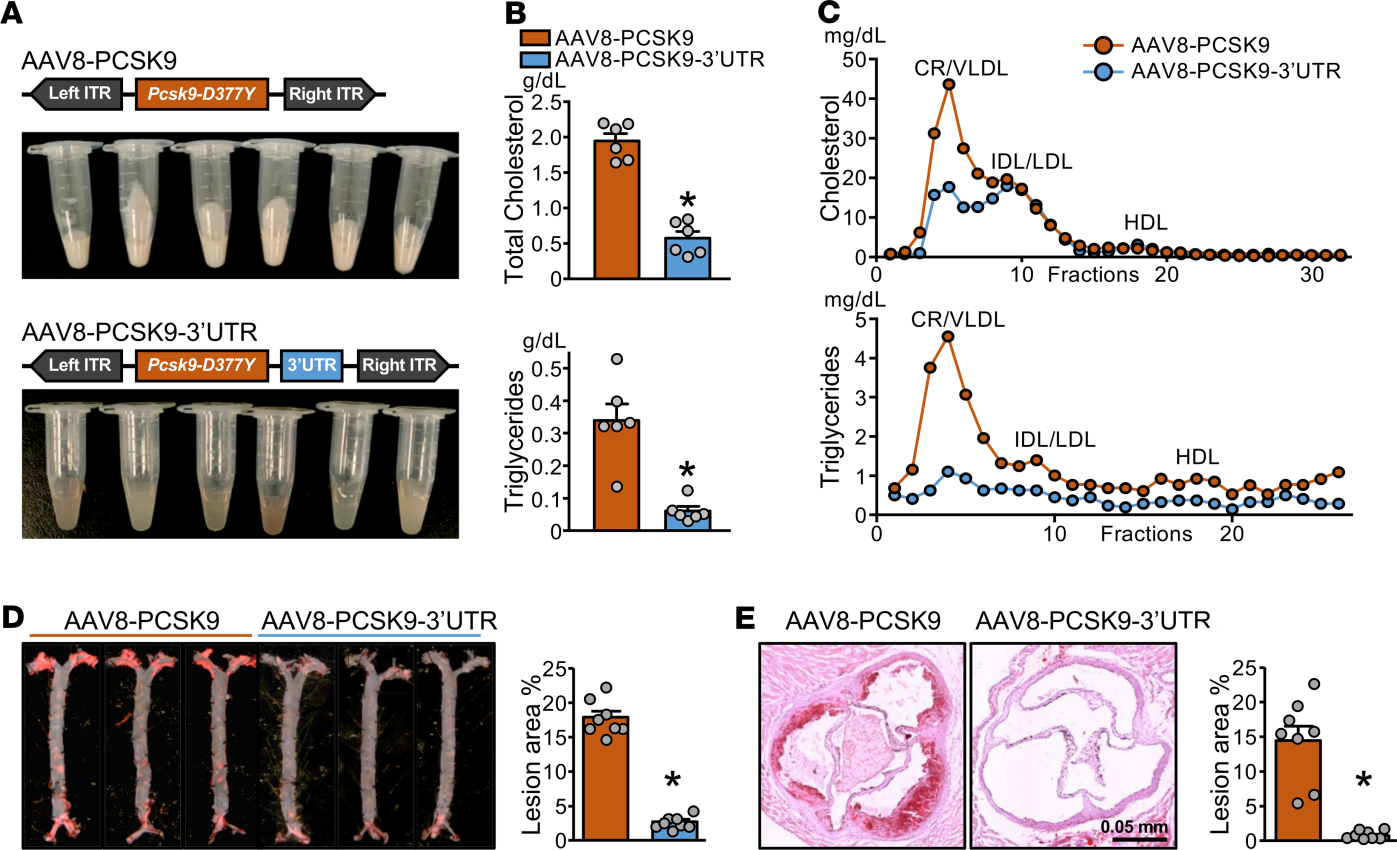
AAV8-PCSK9-3′-UTR alleviates hyperlipidemia and atherosclerosis. (**A**–**E**) Male and female C57BL/6 mice were administered a single dose of AAV8-PCSK9 (w/o 3′-UTR) or AAV8 PCSK9-3′-UTR (with 3′-UTR) via tail vein injection (*n* = 8 in each group) and fed an HFD for 12 weeks before killing. The gross appearance of mouse serum (**A**); serum levels of total cholesterol and triglycerides (**B**)**;** FPLC detection of VLDL, LDL, and HDL (**C**); and representative Oil-red O staining of en face aortae (**D**) and aortic roots (**E**) are shown (original magnification, ×6; scale bars: 0.5 mm). In **B**, **D**, and **E**, data are mean ± SEM from 6–8 mice per group. Normally distributed data were analyzed by 2-tailed Student’s *t* test with Welch correction between 2 groups. *P < 0.05 vs. AAV8-PCSK9. AAV8, adeno-associated virus 8; PCSK9, proprotein convertase subtilisin/kexin type 9; AAV8-PCSK9-3′-UTR, AAV8-based recombinant virus encoding mouse *Pcsk9* mRNA encompassing its native 3′-UTR; HFD, high-fat diet; FPLC, fast protein liquid chromatography; VLDL, very LDL-cholesterol.

**Figure 2:**
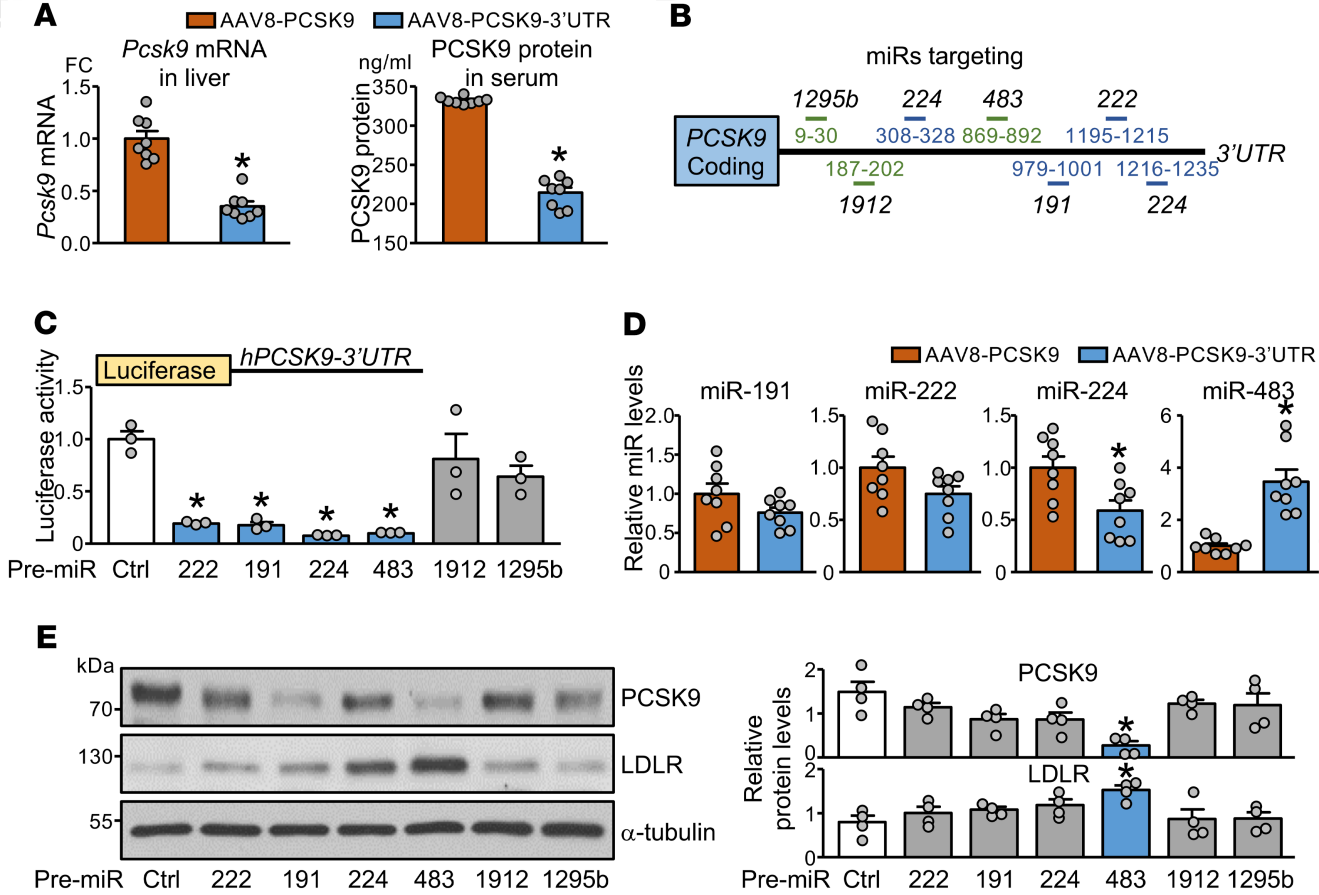
miR-483 regulates PCSK9 via posttranslational mechanism. (**A** and **D**) The animals were used as described in Figure 1. The hepatic *Pcsk9* mRNA levels (**A**), ELISA detection of serum levels of PCSK9 (**A**), and hepatic levels of miR-191, -222, -224, and -483 (**D**) are shown. (**B**) Previously reported (blue lines) or newly predicted (green lines) miRs that bind to the h*PCSK9*-3′-UTR are shown. (**C** and **E**) HepG2 cells were transfected with pre–miR-222 mimic (222), pre–miR-224 mimic (224), pre–miR-483 mimic (483), pre–miR-191 mimic (191), pre–miR-1912 mimic (1912), pre–miR-1295b mimic (1295b), or scramble miR control (Ctrl). In **C**, cells were cotransfected with Luc-PCSK9-3′-UTR reporter. Luciferase activity was measured with pRL-TK activity as a transfection control. In **E**, protein levels of PCSK9 and LDLR were determined by Western blot analysis; α-tubulin was a loading control. In **A** and **D**, data are mean ± SEM from 6–8 mice per group. Normally distributed data were analyzed by 2-tailed Student’s *t* test with Welch correction between 2 groups. In **C** and **E**, data are mean ± SEM from 3–4 independent experiments. Non-normally distributed data were analyzed using Mann-Whitney *U* test between indicated group and control. *P < 0.05 vs. AAV8-PCSK9 or Ctrl. miR, microRNA; PCSK9, proprotein convertase subtilisin/kexin type 9; HepG2, human hepatocellular carcinoma; LDLR, LDL receptor.

The exogenously administered adeno-associated virus 8 (AAV8)-PCSK9 encoding a gain-of-function *Pcsk9* mutant (D377Y) lacking the 3′-UTR region is commonly used to induce hyperlipidemia and experimental atherosclerosis in mouse models (12). To explore whether the 3′-UTR region of the *PCSK9* transcript participates in PCSK9 regulation at the posttranscriptional level, we constructed an AAV8-based recombinant virus encoding mouse *Pcsk9* mRNA encompassing its native 3′-UTR (hereafter called AAV8-PCSK9-3′-UTR). Mice were administered AAV8-PCSK9 or AAV8-PCSK9-3′-UTR, then fed a high-fat diet (HFD) for 12 weeks. The HFD-induced hyperlipidemia was significantly alleviated in mice receiving AAV8-PCSK9-3′-UTR compared with AAV8-PCSK9 (Figure 1A). Consistently, serum levels of total cholesterol, triglycerides, very LDL-C (VLDL-C), and LDL-C and atherosclerosis were reduced in mice with AAV8-PCSK9-3′-UTR (Figure 1, B–E). Also, hepatic *Pcsk9* mRNA levels and serum levels of PCSK9 were decreased in mice with AAV8-PCSK9-3′-UTR (Figure 2A). These results suggest that the 3′-UTR region of *Pcsk9* mRNA exerts an endogenous mechanism against HFD-induced hyperlipidemia and atherosclerosis. To explore whether miR-dependent 3′-UTR targeting accounts for this atheroprotective mechanism, we used bioinformatics to profile miRs that likely target the *PCSK9* mRNA 3′-UTR: miR-222, -191, and -224 were previously reported by others (17), whereas miR-483, -1912, and -1295b were newly predicted by us to target the *PCSK9* mRNA 3′-UTR (Figure 2B and Supplemental Figure 2). A luciferase reporter fused with the *PCSK9*-3′-UTR was constructed to test the efficacy of miRs targeting the *PCSK9*-3′-UTR. Overexpression of miR-222, -191, -224, and -483 but not -1912 or -1295b greatly decreased the luciferase activity (Figure 2C). Among these miRs, only miR-483 increased significantly in the liver with AAV8-PCSK9-3′-UTR compared with AAV8-PCSK9 (Figure 2D). Such increased miR-483 levels, but not those of others, in the liver suggest that miR-483 would contribute to this posttranscriptional regulation of PCSK9 in vivo. Indeed, when validated by Western blot, miR-483 overexpression had the greatest effect on decreasing PCSK9 levels, with attendant increase in LDLR expression (Figure 2E).

### miR-483-5p targeted PCSK9 in hepatocytes.

**Figure 3:**
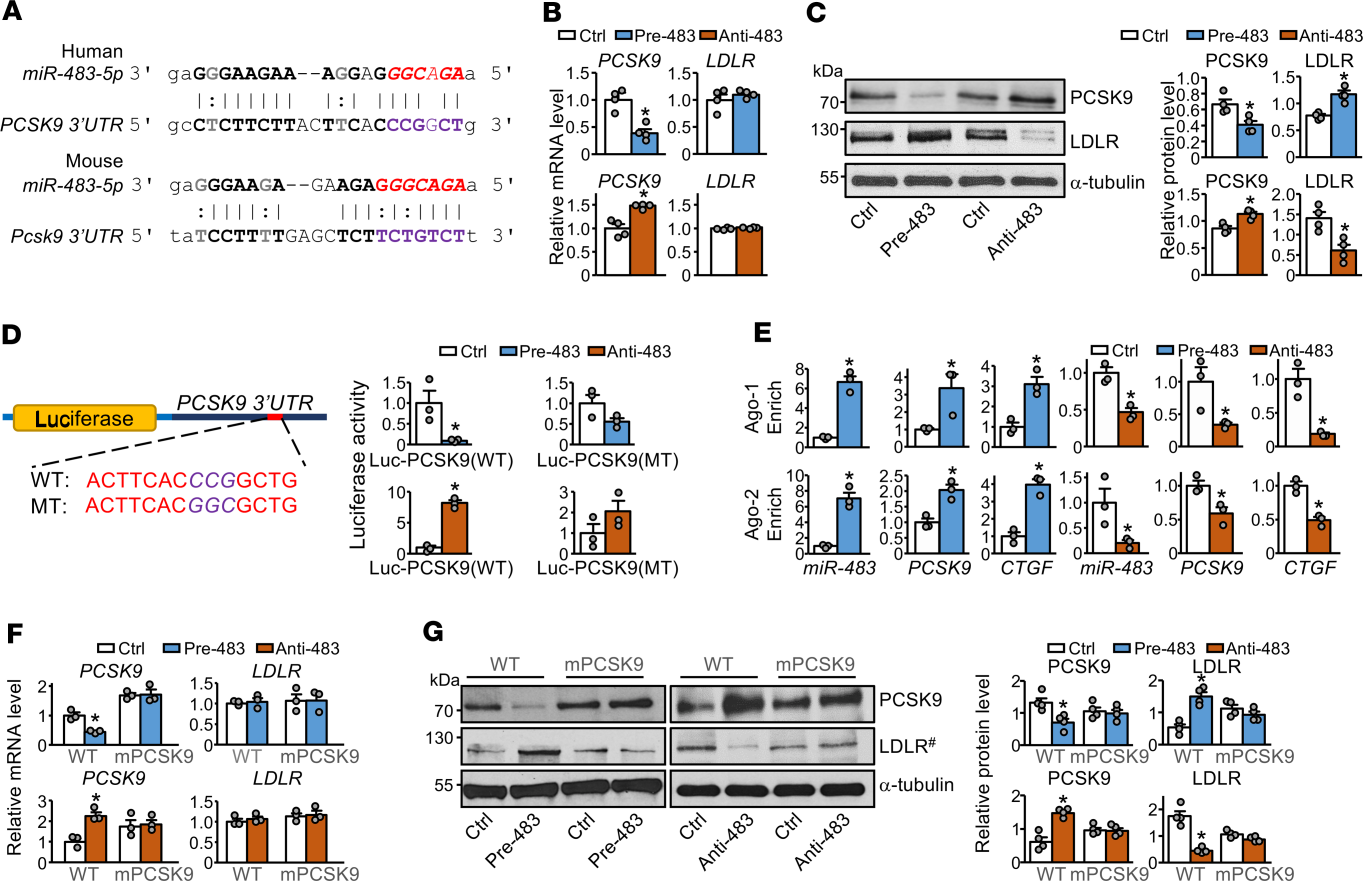
miR-483 targets *PCSK9* and enhances LDLR expression in hepatocytes. (**A**) Bioinformatics prediction of miR-483-5p binding sites in the 3′-UTR of human and mouse *PCSK9* mRNA. In **B**–**G**, HepG2 cells were transfected with pre–miR-483 mimic (pre-483) or anti–miR-483 (anti-483) for 24 hours. (**B** and **C**) mRNA and protein levels of PCSK9 and LDLR. (**D**) HepG2 cells transfected with pre-483 or anti-483 were cotransfected with Luc-PCSK9-3′-UTR (WT) or Luc-mutated PCSK9-3′-UTR (MT). Luciferase activity was measured with pRL-TK activity as a transfection control. (**E**) Ago-1 or Ago-2 immunoprecipitation was performed, and miRISCs-associated miR-483, *PCSK9*, and *CTGF* mRNA levels were quantified by qPCR. (**F** and **G**) mRNA and protein levels of PCSK9 and LDLR in WT HepG2 and mPCSK9 HepG2 cells were transfected with pre-483 or anti-483. ^#^LDLR in the same samples were detected in parallel in a separate gel (**G**). Data are mean ± SEM from 3–4 independent experiments. In **B**–**G**, non-normally distributed data were analyzed using Mann-Whitney *U* test between indicated group and control. *P < 0.05 vs. control. miR, microRNA; PCSK9, proprotein convertase subtilisin/kexin type 9; HepG2, human hepatocellular carcinoma; LDLR, LDL receptor; Luc-PCSK9-3′-UTR (WT), WT *PCSK9* 3′-UTR; Luc-mutated PCSK9-3′-UTR (MT), mutant *PCSK9* 3′-UTR; miRISCs, miRNA-induced silencing complexes; *CTGF*, connective tissue growth factor.

Given that miR-483 might directly target the *PCSK9* 3′-UTR, we used bioinformatics to locate the binding site of miR-483-5p at the 3′-UTR of both human and mouse *PCSK9* mRNA (Figure 3A). A gain-of-function approach involving pre–miR-483 overexpression reduced the mRNA and protein levels of PCSK9 (Figure 3, B and C). In the complementary loss-of-function experiment, HepG2 cells were transfected with an anti–miR-483 construct. The inhibition of miR-483 increased PCSK9 mRNA and protein levels (Figure 3, B and C). Consistently, LDLR expression was increased in HepG2 cells transfected with pre–miR-483 and decreased in those transfected with anti–miR-483. As expected, these changes in LDLR expression were only at the protein level and not the mRNA level (Figure 3, B and C).

To investigate whether miR-483-5p directly targets the predicted binding site at the *PCSK9* 3′-UTR mRNA, we used a luciferase reporter fused to the WT *PCSK9* 3′-UTR [Luc-PCSK9 (WT)] or mutant *PCSK9* 3′-UTR [Luc-PCSK9 (MT)] with mutation of the miR-483-5p targeting site. Pre–miR-483 significantly decreased the luciferase activity of the cotransfected Luc-PCSK9 (WT) in HepG2 cells but only moderately decreased it in cells cotransfected with Luc-PSCK9 (MT) (Figure 3D). However, anti–miR-483 treatment significantly increased the reporter activity of Luc-PCSK9 (WT) but not Luc-PSCK9 (MT). Additionally, in HepG2 cells overexpressing pre–miR-483, both miR-483-5p and *PCSK9* mRNA were enriched in the miRNA-induced silencing complexes (miRISCs) that contained Argonaute-1 or Argonaute-2 (Figure 3E). As a positive control, the mRNA level of *CTGF*, an established miR-483 target (18), was also increased in the miRISCs. The sequestration of *PCSK9* and *CTGF* mRNA in miRISCs was decreased in HepG2 cells transfected with anti–miR-483.

To further confirm miR-483 targeting of *PCSK9*, we used CRISPR/Cas9 gene editing to create a HepG2 mutant cell line (mPCSK9 HepG2) with deletion of the miR-483 targeting site in the *PCSK9* 3′-UTR (Supplemental Figure 3). Transfection of pre–miR-483 or anti–miR-483 did not significantly change the mRNA or protein levels of PCSK9 or LDLR in mPCSK9 HepG2 cells (Figure 3, F and G).

### miR-483-5p increased LDL uptake in hepatocytes.

**Figure 4:**
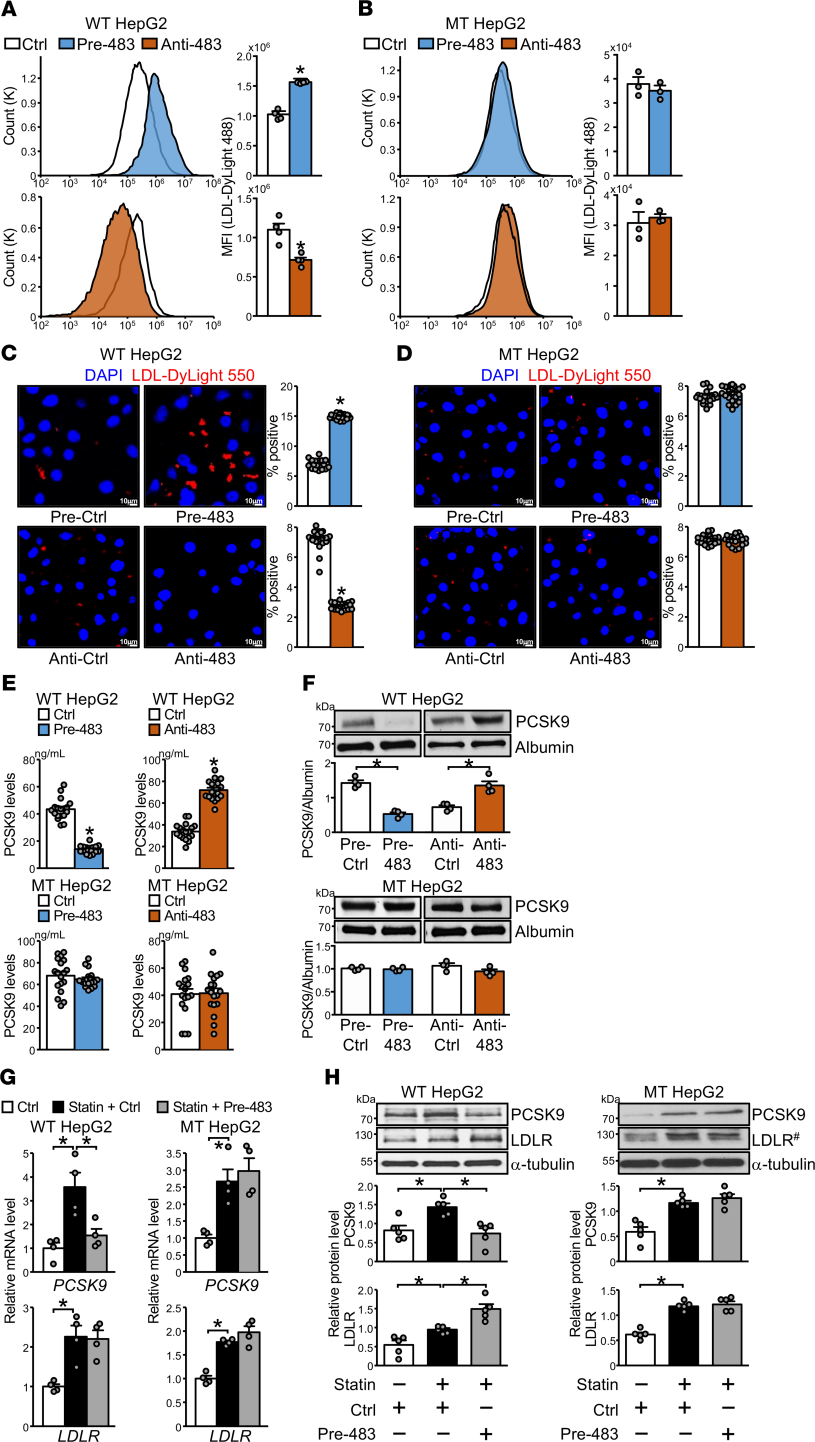
miR-483 overexpression in HepG2 cells increases LDL-C uptake. (**A**–**H**) HepG2 and mPCSK9 HepG2 cells were transfected with pre-483 or anti-483 as indicated. Fluorescent-labeled LDL was incubated with HepG2 and mPCSK9 HepG2 cells. LDL uptake was detected by flow cytometry (**A** and **B**) or confocal microscopy (**C** and **D**) (original magnification, ×20; scale bars: 10 μm). (**E** and **F**) Levels of PCSK9 in conditioned media were measured by ELISA and Western blot analysis. (**G** and **H**) HepG2 and mPCSK9 HepG2 cells were incubated with 1 μM atorvastatin for 24 hours. mRNA and protein levels of PCSK9 and LDLR were determined by qPCR and Western blot analysis. In MT HepG2 cells, ^#^LDLR in the same samples were detected in parallel in a separate gel (**H**). Data are mean ± SEM from at least 4 independent experiments. In **A**, **B**, and **F**, non-normally distributed data were analyzed using Mann-Whitney *U* test between 2 groups. In **C**–**E**, normally distributed data were analyzed by 2-tailed Student’s *t* test with Welch correction between 2 groups. In **G** and **H**, non-normally distributed data were analyzed using Kruskal-Wallis test with Dunn’s multiple comparisons between indicated groups. *P < 0.05 vs. Ctrl or between 2 indicated groups. miR, microRNA; HepG2, human hepatocellular carcinoma; LDL-C, LDL-cholesterol; PCSK9, proprotein convertase subtilisin/kexin type 9.

The functional consequence of miR-483 targeting *PCSK9* was evaluated by testing its impact on LDLR-mediated LDL clearance. Overexpression of pre–miR-483 in HepG2 cells significantly increased the binding of fluorescence-labeled LDL, as assessed by flow cytometry and immunostaining (Figure 4, A and C). The miR-483–mediated increase in LDL binding was absent in mPCSK9 HepG2 cells (Figure 4, B and D). Transfection of anti–miR-483 had the opposite effect and decreased LDL binding in HepG2 cells but had no impact on LDL binding in mPCSK9 HepG2 cells (Figure 4, A–D). Mature PCSK9 is secreted from cultured hepatocytes to the conditioned media. As expected, overexpression of pre–miR-483 decreased and anti–miR-483 transfection increased PCSK9 levels in conditioned media, as revealed by ELISA (Figure 4E, top) and Western blot analysis (Figure 4F, top). However, neither pre–miR-483 nor anti–miR-483 altered PCSK9 secretion in medium from mPCSK9 HepG2 cells (Figure 4E, bottom, and Figure 4F).

Part of a counterregulatory effect of statins is the SREBP2-mediated induction of both LDLR and PCSK9 in hepatocytes, which in turn decreases LDLR expression (3, 11). We tested whether miR-483-5p could reverse this unwanted effect of statins. Pre–miR-483 transfection in HepG2 cells decreased the atorvastatin-conferred increase in PCSK9 mRNA and protein levels (Figure 4, G and H). Importantly, this reduction in PCSK9 levels with combined atorvastatin and pre–miR-483 further increased LDLR expression compared with atorvastatin alone; this effect of miR-483-5p on rectifying LDLR expression was not observed in mPCSK9 HepG2 cells (Figure 4, G and H). Taken together, data in Figure 3 and Figure 4 suggest that miR-483-5p directly targets the 3′-UTR of *PCSK9* mRNA in cultured hepatocytes, which results in augmented LDLR expression and increased LDL uptake.

### Decreased circulatory levels of miR-483 in hyperlipidemic mice and humans.

**Figure 5:**
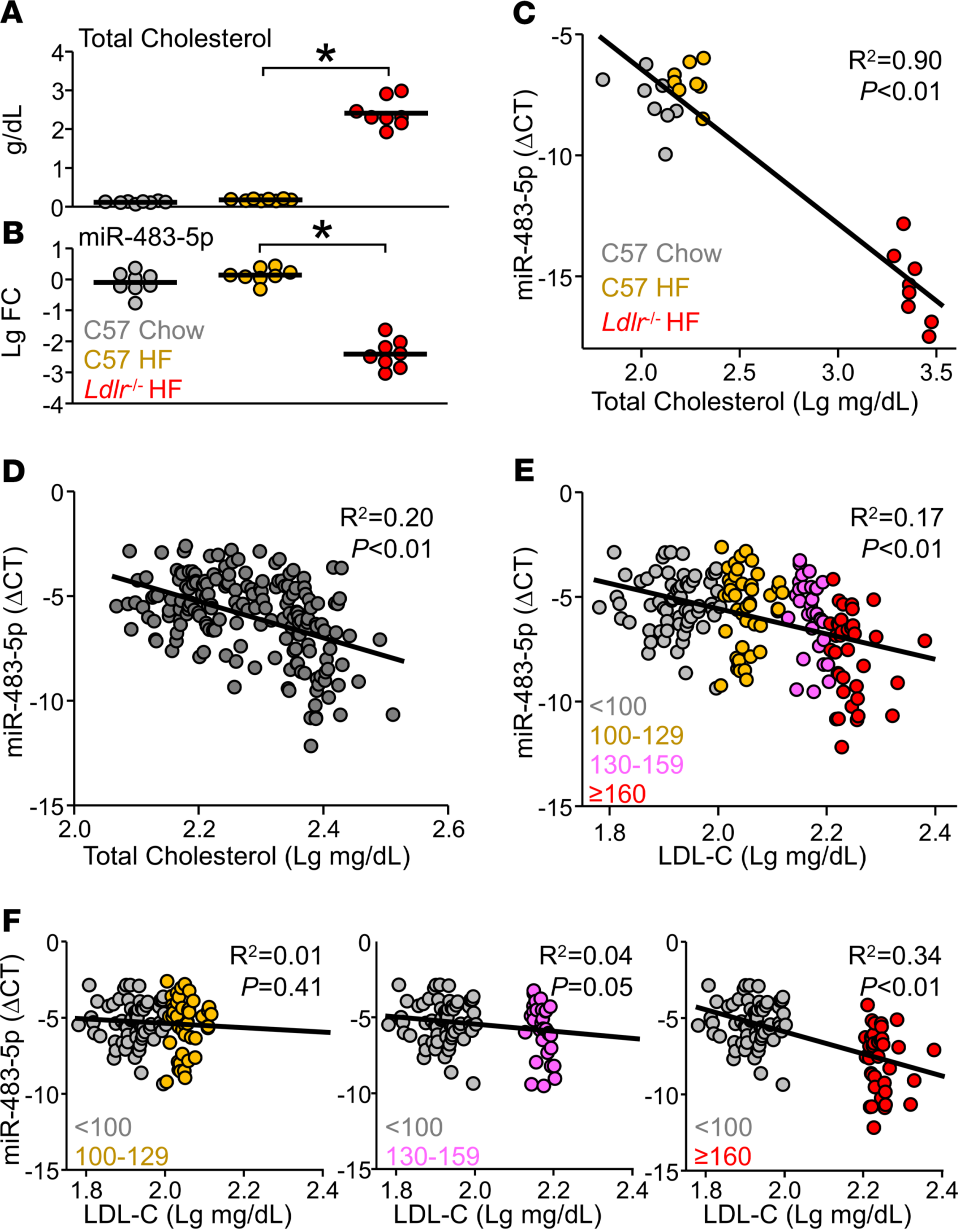
miR-483 levels were decreased in hyperlipidemic mice and human subjects. (**A** and **B**) Male and female C57BL/6 mice were fed an HFD or chow diet, and *Ldlr*^-/-^ mice were fed an HFD for 6 weeks. (**C**) Correlation of serum levels of miR-483-5p and total cholesterol assessed by the Pearson correlation. (**D**) Serum was collected from humans (*n* = 179). Serum levels of total cholesterol and miR-483-5p were measured and correlated. (**E** and **F**) Humans (*n* = 179) were divided into 4 groups based on LDL-C levels: <100 mg/dL (optimal, *n* = 65, gray dots); 100–129 mg/dL (near/above optimal, *n* = 46, orange dots); 130–159 mg/dL (borderline high, *n* = 31, pink dots); and ≥160 mg/dL (high, *n* = 37, red dots). The correlation between serum levels of LDL-C and miR-483-5p is shown in **E**. Correlations between the optimal group with the other 3 subgroups (*n* = 111, 96, 102, respectively) are shown in **F**. ΔCT represents the difference between the cycle threshold of miR-483-5p and Cel-miR-39. In **A** and **B**, data are mean ± SEM from 8 mice per group. Normally distributed data were analyzed by 1-way ANOVA test with a Bonferroni’s post hoc test between 2 indicated groups. The correlation analysis was assessed by the Pearson method. *P < 0.05. miR, microRNA; HFD, high-fat diet; LDL-C, LDL-cholesterol.

Because miR-483, a secretory miR, and circulatory levels of PCSK9 are increased in hyperlipidemic rodent models and human subjects (18, 20, 22), we investigated whether hyperlipidemia is associated with reduced levels of miR-483 in circulation. Initially, we compared the circulatory levels of miR-483-5p and total cholesterol among 3 mouse groups: C57BL/6 mice fed an HFD or chow diet and *Ldlr*-knockout mice fed an HFD. Total cholesterol levels were greatly increased and serum levels of miR-483-5p were significantly reduced in *Ldlr*-knockout mice fed an HFD compared with WT mice fed an HFD or chow diet (Figure 5, A and B). Pearson’s correlation analysis showed an inverse correlation between serum levels of miR-483 and total cholesterol in these mice (*P* < 0.01, *R*^2^ = 0.90, *n* = 24) (Figure 5C). With these results from mouse models, we next explored whether this inverse correlation existed in a cohort of 179 humans without a diagnosis of cardiovascular disease. The serum levels of miR-483-5p and total cholesterol were indeed inversely correlated among these individuals (*P* < 0.01, *R*^2^ = 0.20) (Figure 5D). To this end, we separated these individuals into 4 groups based on their LDL-C levels (i.e., <100 mg/dL [optimal]; 100–129 mg/dL [near/above optimal]; 130–159 mg/dL [borderline high]; ≥160 mg/dL [high]; ref. 23).

Compared with individuals with LDL-C less than 100 mg/dL, for the other 3 groups, the serum levels of miR-483-5p were significantly lower (*P* = 0.699 vs. near/above optimal, 0.196 vs. borderline high, and < 0.0001 vs. high) (Supplemental Figure 4). Consistent with the correlation shown in Figure 5D, the serum levels of LDL-C and miR-483-5p were inversely correlated (Figure 5E). When comparing the group with LDL-C less than 100 mg/dL (optimal) with the other 3 groups (near/above optimal; borderline high; high), the larger difference in LDL-C levels, the inverse correlation of miR-483-5p levels was more significant (Figure 5F). Together, these data from mouse models and humans suggest that the serum levels of miR-483 were inversely correlated with hyperlipidemia.

### miR-483 reduced LDL-C levels by targeting hepatic Pcsk9 in mouse models.

**Figure 6:**
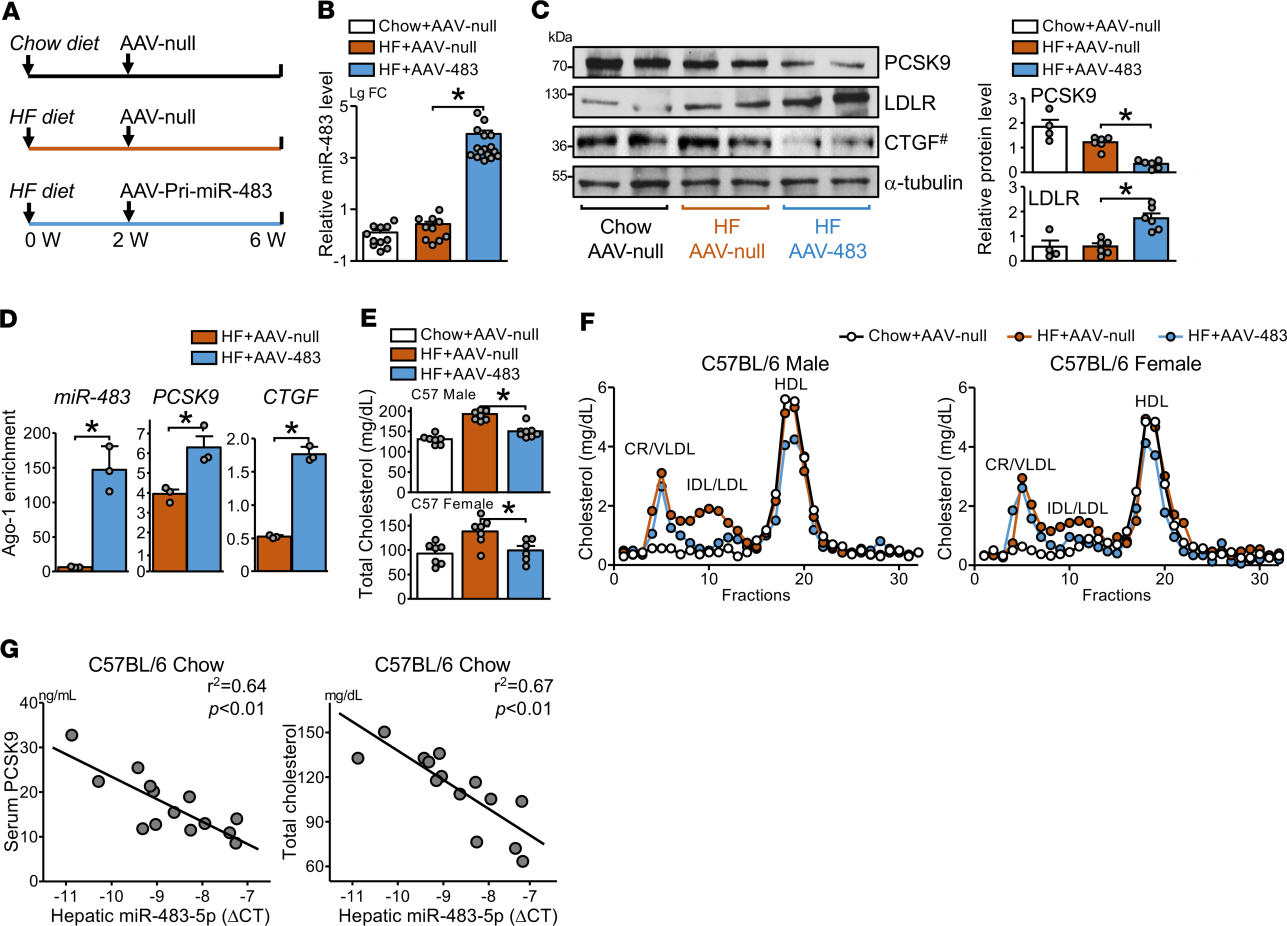
miR-483 reduces circulatory levels of LDL-C in mice. (**A**) Male and female C57BL/6 mice were fed an HFD or chow diet for 6 weeks. A single dose of AAV8-null (AAV-null) or AAV8-pri-miR-483 (AAV-483) was administered by tail vein injection at the end of week 2. (**B** and **C**) Hepatic miR-483-5p levels were determined by qPCR, protein levels of PCSK9, LDLR, and CTGF were detected by Western blot. ^#^CTGF in the same samples were detected in parallel in a separate gel (**C**). (**D**) Ago-1 was immunoprecipitated from fixed liver tissue, and Ago1-associated miR-483-5p, *Pcsk9*, and *Ctgf* mRNA levels were quantified by qPCR. (**E**) Total cholesterol levels measured by cholesterol assay. (**F**) Serum levels of VLDL, LDL, and HDL were determined by FPLC. (**G**) The correlations between hepatic miR-483-5p expression levels and serum levels of PCSK9 (left) or total cholesterol (right) are shown. ΔCT represents the difference between the cycle threshold of miR-483-5p and U6. The numbers of mice used are shown in Supplemental Table 1. Data are mean ± SEM. In **B**, non-normally distributed data were analyzed using Kruskal-Wallis test with Dunn’s multiple comparisons between indicated groups. In **C** and **D**, non-normally distributed data were analyzed using Mann-Whitney *U* test. In **E**, normally distributed data were analyzed by 1-way ANOVA test with a Bonferroni’s post hoc test between 2 indicated groups. In **G**, the correlation analysis was assessed by the Pearson method. *P < 0.05. miR, microRNA; LDL-C, LDL-cholesterol; HFD, high-fat diet; AAV8, adeno-associated virus 8; PCSK9, proprotein convertase subtilisin/kexin type 9; LDLR, LDL receptor; CTGF, connective tissue growth factor; VLDL, very LDL; IDL, intermediate-density lipoprotein.

Results from Figures 1–5 led us to investigate whether exogenously administered miR-483 can alleviate HDF-induced hyperlipidemia in mouse models. We used AAV8-infected pri–miR-483 (hereafter called AAV-483) to achieve hepatic overexpression of miR-483. Male and female C57BL/6 mice administered AAV-483 or parental empty AAV8 vector (AAV-null) were fed an HFD for 6 weeks. A group of mice administered AAV-null and fed a chow diet was a baseline control (Figure 6A). Hepatic miR-483-5p levels were significantly elevated in mice receiving AAV-483 versus AAV-null (Figure 6B). Moreover, the efficacy of hepatic delivery of miR-483 by AAV-483 was evidenced by much higher miR-483 levels in the liver than other nontarget tissues such as kidney, lung, and heart (Supplemental Figure 5). As anticipated, PCSK9 protein levels were decreased and LDLR protein levels were increased in the livers of animals receiving AAV-483 (Figure 6C). As a positive control, CTGF protein levels were decreased in the livers of these animals (Figure 6C). In line with the results that miR-483 targeted *PCSK9* and *CTGF*, mRNA levels of *Pcsk9* and *Ctgf* were enriched in miRISCs isolated from the livers of mice receiving AAV-483 versus AAV-null (Figure 6D). The HFD-induced hypercholesterolemia was evident from the lipoprotein profiles in male and female mice administered AAV-null, as measured by colorimetric assay and fast protein liquid chromatography (FPLC) (Figure 6, E and F, and Supplemental Table 1). AAV-483 administration reduced the HFD-induced hypercholesterolemia by reducing the levels of circulating IDL and LDL without affecting VLDL or HDL-associated cholesterol (Figure 6, E and F). Of note, the serum levels of PCSK9 and total cholesterol were inversely correlated with liver miR-483-5p expression (Figure 6G). We reasoned that the mechanism by which miR-483 decreases LDL-C levels depends on its suppression of the PCSK9–LDLR axis. Thus, as negative controls, male and female *Ldlr*-knockout mice were fed an HFD and administered AAV-483 or AAV-null. Although AAV-483 greatly increased miR-483-5p levels and blunted the PCSK9 and CTGF levels in the livers of *Ldlr*-knockout mice (Supplemental Figure 6, A and B), the levels of total cholesterol and LDL-C were comparable between mice administered AAV-483 or AAV-null (Supplemental Figure 6, C and D, and Supplemental Table 1).

### miR-483 required Pcsk9 3′-UTR to reduce LDL-C levels in vivo.

**Figure 7:**
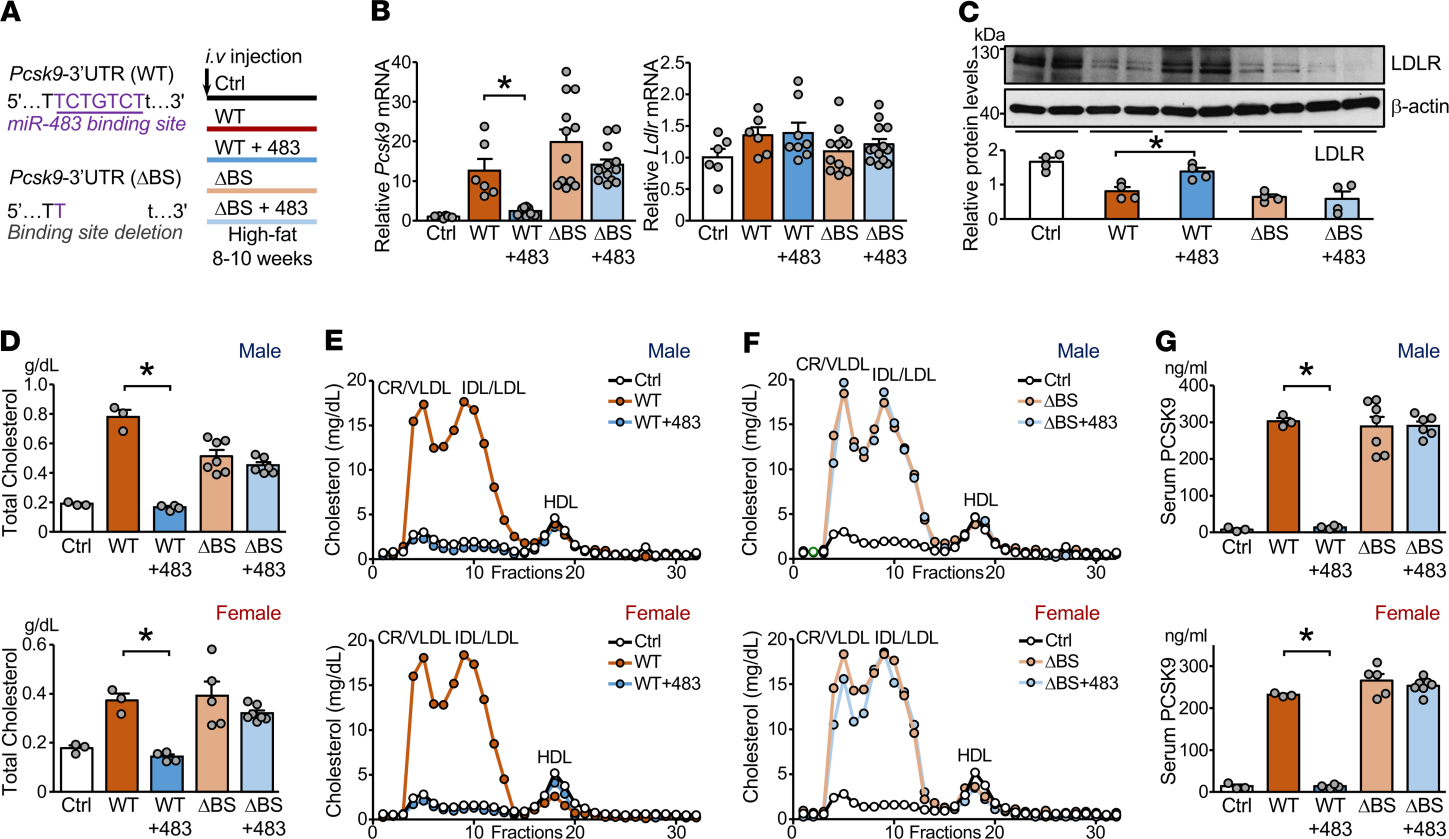
miR-483 targets *Pcsk9* 3′-UTR to reduce LDL-C levels in mice. (**A**) Male and female C57BL/6 mice were administered AAV8-PCSK9-3′-UTR WT (WT) or AAV8-PCSK9-3′-UTR with a deleted miR-483 binding site (ΔBS) together with AAV-miR-483 or AAV-null by tail vein injection (*n* = 6–13 in each group). All mice were fed an HFD for 8–10 weeks. (**B**) Levels of *Pcsk9* and *Ldlr* mRNA in mouse liver were determined by qPCR. (**C**) Protein levels of hepatic LDLR were detected by Western blot analysis. (**D**–**F**) Serum levels of total cholesterol, VLDL, LDL, and HDL are shown. (**G**) Serum levels of PCSK9 assessed by ELISA are shown. The numbers of mice used are shown in Supplemental Table 1. Data are mean ± SEM. In **B**, normally distributed data was analyzed by 1-way ANOVA test with a Bonferroni’s post hoc test between 2 indicated groups. In **C**, **D**, and **G**, non-normally distributed data were analyzed using Mann-Whitney *U* test between 2 indicated groups. *P < 0.05 vs. WT. miR, microRNA; LDL-C, LDL-cholesterol; AAV8, adeno-associated virus 8; PCSK9, proprotein convertase subtilisin/kexin type 9; AAV8-PCSK9-3′-UTR, AAV8-based recombinant virus encoding mouse *Pcsk9* mRNA encompassing its native 3′-UTR; HFD, high-fat diet; LDLR, LDL receptor; VLDL, very LDL.

To further study the role of the *Pcsk9* 3′-UTR in miR-483 targeting, we administered male or female C57BL/6 mice with AAV8-PCSK9-3′-UTR WT (hereafter called AAV-3′-UTR WT) encoding the coding region of mouse *Pcsk9* fused with the 3′-UTR or AAV8-PCSK9-3′-UTR with the deleted miR-483 binding site (hereafter called AAV-3′-UTR ΔBS). All groups were fed an HFD for 8 to 10 weeks and treated in parallel with or without AAV-483 or AAV-null (Figure 7A). Only mice receiving AAV-3′-UTR WT together with AAV-483 showed reduced *Pcsk9* mRNA levels (Figure 7B). *Pcsk9* mRNA levels were least affected in mice receiving AAV-483 and AAV-3′-UTR ΔBS (Figure 7B). Correspondingly, AAV-483 administration increased the expression of LDLR in livers (Figure 7C) and decreased circulating levels of total cholesterol and LDL-C in mice receiving AAV-3′-UTR WT (Figure 7, D and E, and Supplemental Table 1) but not AAV-3′-UTR ΔBS (Figure 7, C–F, and Supplemental Table 1). Phenotypically, mice receiving AAV-3′-UTR WT but not AAV-3′-UTR ΔBS showed decreased serum levels of PCSK9 resulting from miR-483 targeting of *Pcsk9* 3′-UTR (Figure 7G). Together, these data suggest that miR-483 lowers circulating LDL-C levels by directly targeting the 3′-UTR of *Pcsk9*, which suggests the possibility for using miR-483 in treating hyperlipidemia.

## Discussion

This study demonstrates that the exogenously administered miR-483 can substantially reduce total cholesterol and LDL-C levels in hypercholesterolemic mouse models. The underlying mechanism relies on miR-483 inhibiting PCSK9 expression, thereby increasing the hepatocyte expression of LDLR. Mechanistically, miR-483 targeting the 3′-UTR of *PCSK9* mRNA was validated by Luc-PCSK9 reporter assay (Figure 3D) and Ago-IP experiments (Figure 3E and Figure 6D). miR-483 and *PCSK9* mRNA in the miRISCs were also enriched in the photoactivatable-ribonucleoside–enhanced cross-linking and immunoprecipitation data set reported by Hafner et al. (24). miR-483 targeting of the *Pcsk9* 3′-UTR in vivo was validated by comparing the circulatory levels of LDL-C in mice receiving AAV-3′-UTR WT or AAV-3′-UTR ΔBS (Figure 7, D–F).

In the mouse experiments, the amount of miR-483 administered would be above physiological levels. Such supraphysiological doses of miR-483, when given to mice, robustly increase the hepatic and circulatory levels of miR-483. We also conducted experiments with doses as low as 4 × 10^10^ vector genomes of AAV-483, which still showed efficacy in modulating the expression of PCSK9 and LDLR (Supplemental Figure 7). The Further Cardiovascular Outcomes Research with PCSK9 inhibition in Subjects with Elevated Risk study and the ODYSSEY OUTCOME trial showed a greater reduction of LDL-C levels and cardiovascular events by combination therapy with PCSK9 mAb and statins than with statin monotherapy (10, 25, 26). Data in Figure 4, G and H, show that miR-483 directly targeting the 3′-UTR of *PCSK9* mRNA could mitigate the unwanted effect of statins in increasing plasma PCSK9 protein levels. These results demonstrate the potential use of miR-483 together with statins for treating hypercholesterolemia.

PCSK9 mAbs bind to the catalytic site of PCSK9, thereby inhibiting the extracellular interaction of PCSK9 with the EGF-A domain of the LDLR (6). Presumably, miR-483 inhibits PCSK9 via targeting *PCSK9* mRNA intracellularly in hepatocytes. In humans, the plasma level of PCSK9 is positively correlated with the LDL-C level (22). PCSK9 levels were reduced in both the livers and the serum of our mice receiving AAV-483. These results suggest that miR-483 targeting *PCSK9* mRNA in the liver decreased the amount of PCSK9 in circulation, which is consistent with decreased PCSK9 levels in patients receiving evolocumab or alirocumab (27, 28). Although miR-483 may decrease intracellular *PCSK9* mRNA levels in hepatocytes, the PCSK9 mAbs could antagonize PCSK9 in circulation. The synergism of miR-483 and PCSK9 mAbs may have greater cholesterol-lowering effects. Intriguingly, the circulatory levels of miR-483-5p contrasted with LDL-C levels in hyperlipidemic mouse models and humans, and miR-483-5p levels seemed to decrease more with high LDL-C levels (Figure 5). Despite unclear homeostatic ranges and regulatory mechanisms of the circulatory level of miR-483, our results support the use of exogenously administered miR-483 to ameliorate hyperlipidemia.

Besides miR-483, several other miRNAs, including miR-222, -191, and -224, have been predicted to target *PCSK9* mRNA (17). However, the efficacy of these miRNAs in lowering LDL-C levels and attenuating atherosclerosis have not been tested in vitro and in vivo. Besides, results in Figure 2 suggest that miR-483 has a greater potency than other miRs in *PCSK9* targeting. Besides targeting *PCSK9* mRNA, miR-483 likely targets several genes and pathways involved in nonalcoholic fatty liver disease (NAFLD), including *IL1B*, *IL6*, *TGFB1*, and monocyte chemoattractant protein 1 (20). Thus, miR-483 may exert a pleiotropic effect to mitigate hyperlipidemia-associated NAFLD. In support of this thesis, we found that mice fed an HFD had less severe NAFLD when miR-483 was coadministered. This additional beneficial effect of miR-483 was evidenced by the alleviated lipid deposition in the liver, reduced levels of hepatic transaminases in circulation, and attenuated expression of *Il1b*, *Tnfa*, *Tgfb1*, and *Fbn1* (Supplemental Figure 8).

Both miR-483-3p and miR-483-5p are encoded by the *IGF2-miR-483* gene. From bioinformatics prediction, miR-483-5p may target the cognate sequence in the *PCSK9* mRNA better than miR-483-3p. Experimentally, luciferase reporter and LDLR expression assays validated the superiority of miR-483-5p in targeting *PCSK9* transcript (Figure 3, C and D, and Supplemental Figure 9). Thus, for therapeutic efficacy, miR-483-5p delivery should be considered. For miR-based therapeutics, the stability and specificity of miR delivery are 2 major challenges. Compared with the delivery systems used in current miR-related clinical trials (e.g., locked nucleic acids-, N-acetyl-D-galactosamine-, or cholesterol-conjugated miRs), the AAV8-based system features high efficacy for hepatic delivery, but this approach is still limited to experimental animals (29). Recent advancement in nanoparticles for tissue-specific drug delivery may be considered for therapeutic use of miR-483-5p (30, 31). Additionally, optimization of natural miR-483 function with medicinal chemistry approaches may allow for more translational therapeutic approaches in humans (32).

In summary, we found a mechanism by which miR-483-5p increases the hepatic expression of LDLR via targeting *PCSK9* 3′-UTR. The pharmaceutical efficacy of this mechanism relies on miR-483 administration greatly reducing total cholesterol and LDL-C levels in experimental hyperlipidemia.

## Methods

### Cell culture.

Human hepatocellular carcinoma cells (HepG2) were obtained from ATCC (catalog HB-8065). Cells were maintained in DMEM (Gibco) containing 10% fetal bovine serum, 2 mM L-glutamine, 100 U/mL penicillin, and 100 μg/mL streptomycin sulfate at 37°C and 5% CO_2_.

### Bioinformatics prediction of miRNA targeting sites.

Putative miR-483 targeting sites in the 3′-UTR of the *PCSK9* gene were predicted by using miRanda v3.3a with the parameters “-sc 120, -scale 4, -en -10” (33). The predicted binding sites with the highest score were selected for validation.

### miR mimics and anti-miRs transfection.

The miR mimics (pre-miR) and anti-miRs were purchased from Ambion. HepG2 cells at 50%–70% confluence were transfected with 60 nM pre-miR or anti-miR using Lipofectamine 2000 (Invitrogen) in Opti-MEM medium for 6 hours and then changed to complete growth medium. At 24 hours after transfection, cells were lysed. Equal amounts of MirVana miRNA Mimic Negative Control #1 (i.e., Pre-Ctrl) or anti-miR miRNA Inhibitor Negative Control #1 (i.e., Anti-Ctrl) were used as controls for pre-miR or anti-miR experiments, respectively.

### Luciferase reporter plasmids, transfection, and luciferase assay.

The 908-bp human *PCSK9* 3′-UTR containing the miR-483 putative binding site was subcloned (forward primer, CGGACTAGTACTGTGGGGCATTTCACCAT, reverse primer, CGACGCGTGCAACAGAGAGGACAGACCC; restriction enzyme cutting on both ends with SpeI and MluI, respectively) into the pMIR-REPORT vector (Ambion) to generate the pMIR-Luc-PCSK9-3′-UTR WT reporter [Luc-PCSK9 (WT)]. Then the CGG to GCC mutations in the miR-483 binding seed sequence of the *PCSK9* 3′-UTR were introduced into the Luc-PCSK9 (WT) plasmid by using the QuikChange Lightning Multi Site-Directed Mutagenesis Kit (Agilent Technologies). Renilla luciferase plasmid (pRL-TK) was used as the transfection control. Luc-PCSK9 (WT), Luc-PCSK9 (MT), and control pRL-TK plasmids were cotransfected into HepG2 cells by using Lipofectamine 2000 (Invitrogen). At 24 hours after transfection, cells were lysed for luciferase activity measurement with the Dual-Glo Luciferase Reporter Assay Kit (Promega).

### Ago1 and Ago2 immunoprecipitation.

HepG2 cells were cross-linked under 400 mJ/cm^2^ UV light, then lysates were incubated with protein G Dynabeads conjugated with anti-Ago1 or anti-Ago2 antibody (Wako Chemicals) at 4°C overnight. The same amount of mouse IgG was used as an isotype control. The immunoprecipitated RNAs and input RNAs were extracted by using Trizol for qPCR detection.

### AAV8 hepatic-specific overexpression vectors.

pAAV8/D377Y-mPCSK9 plasmid was obtained from Addgene. Mouse *Pcsk9* 3′-UTR was subcloned into the FseI site (WT, AAV8-PCSK9-3′-UTR WT), then the 5′-CTGTCT-3′ miR-483 binding site was deleted (ΔBS, AAV8-PCSK9-3′-UTR ΔBS) as shown in Figure 7A. AAV8-miR-483 was constructed with the *Pcsk9* coding sequence replaced by mouse pri-mR-483 between AgeI and FseI sites. AAVs and empty control viruses were enveloped by the UCSD Vector Development Core. Various AAV8 viruses (1 × 10^12^ vector genomes) were administered to mice by tail vein injection.

### CRISPR/Cas9–mediated miR-483 binding-site disruption.

Single-guide RNAs were designed by using an online tool (34) (Supplemental Figure 3). Oligonucleotide pairs with BbsI-compatible overhangs were annealed and cloned into the vector pX330-U6-Chimeric_BB-CBh-hSpCas9 (Addgene). The XhoI restriction enzyme site was then introduced into the 5′ end of the inserted gRNA and subcloned with SnaBI in the 3′ end. The product including the gRNA and gRNA scaffold was inserted into the Adeno Cas9 plasmid (Addgene) for virus production. Adenovirus and empty control virus were enveloped by the UCSD Vector Development Core. HepG2 cells were infected with the produced adenovirus or empty control virus at 3 × 10^6^ plaque-forming units to each well of a 6-well plate for 2 days. The mixed population of infected HepG2 cells was selected for genotyping.

### RT-qPCR and Western blot analysis.

RNA was isolated from cultured cells, liver tissues, or serum by using TRIzol or TRIzol LS (Invitrogen). Total RNA was reverse-transcribed with use of the PrimeScript RT-PCR Kit (Takara), followed by qPCR with SYBR Green (Bio-Rad) in a Bio-Rad CFX-96 real-time system. Primers used in this study are in Supplemental Table 2. The relative mRNA level was calculated by the 2^(-ΔΔCq)^ method with β-actin as an internal control. For miR quantification, the TaqMan MicroRNA Assay followed the manufacturer’s protocol (Life Technologies). U6 was detected as the internal control in cultured cells and tissues from animals. To quantify the miR-483 level in serum, a *C*. *elegans* miR (i.e., Cel-miR-39) was spiked in at 2 nM before RNA extraction, with the level used as the internal control.

Protein extracts from cultured cells or tissues were resolved by SDS-PAGE and transferred to 0.45 μm NC membranes. Western blot analysis was performed with the following antibodies: anti-PCSK9 (Abcam, catalog ab28770), anti-LDLR (Abcam, catalog ab30532), anti-CTGF (Abcam, catalog ab6992), anti–α-tubulin (Cell Signaling, catalog 2144), and anti–β-actin antibody (Santa Cruz Biotechnology, catalog sc-47778). Horseradish peroxidase-conjugated anti-rabbit (Cell Signaling, catalog 7074), anti-mouse (Cell Signaling, catalog 7076), or anti-goat (Dako, catalog P0449) were used as secondary antibodies.

### LDL uptake assay.

Monolayers of HepG2 cells were incubated with 50 μg/mL fresh, isolated, nonacetylated human LDL labeled with Dil (Invitrogen) for 1 hour in the dark with serum-free medium. Cells were trypsinized to obtain a single-cell suspension and Dil-LDL uptake was quantified by flow cytometry (BD Accuri C6 flow cytometer). LDL Uptake Assay Kit (Cayman) was used to evaluate LDL uptake. Briefly, cells were incubated with 5 μg/mL LDL-DyLight 550 working solution prepared in serum-free medium for 12 hours, then fixed and imaged under a fluorescence microscope (Olympus FV1000 Spectral Confocal).

### Animal experiments.

C57BL/6J mice (stock no. 000664) and *Ldlr^-/-^* mice (stock no. 002207) obtained from the Jackson Laboratory were maintained under a specific pathogen–free environment (21°C ± 2°C, 50% ± 10% relative humidity, and 12-hour light/12-hour dark cycle).

Mice fed an HFD or chow diet were administered AAV8-null or AAV8-miR-483 with or without AAV8 PCSK9-3′-UTR (WT) or AAV8 PCSK9-3′-UTR (ΔBS) by tail vein injection. After 6–12 weeks, animals were killed, and blood was collected from the left ventricle by using a heparinized syringe fitted with a 25-gauge needle. Serum levels of liver aspartate aminotransferase and alanine transaminase were determined by using the Aspartate Aminotransferase (AST) and Alanine Transaminase (ALT) Detection Kits (Teco Diagnostics). For all experiments, both sexes of mice with the same age and similar weight were randomly assigned to experimental and control groups. Frozen sections of livers and hearts were cut 5 μm thick and stained with 0.5% Oil-red O, then counterstained with hematoxylin. The entire aortic trees were opened longitudinally, stained with Oil-red O, and digitally scanned. Histological analysis and image processing involved use of the Hamamatsu Nanozoomer 2.0HT Slide Scanner. En face lesion area was assessed with WinROOF 6.5 (Mitani Co).

### ELISA.

PCSK9 levels in cell culture medium and serum were measured with a human or mouse PCSK9 ELISA Kit (R&D Systems) according to the manufacturer’s protocols. Briefly, cell culture supernatants or serum samples were diluted and incubated in precoated plates for 2–4 hours. After 2 hours of incubation with horseradish peroxidase–conjugated secondary antibody, TMB substrate solution was added and samples were incubated further for 30 minutes before stop buffer was added. Plates were read immediately by using a microplate reader (BioTeK) set at 450 nM.

### Measurement of lipoprotein profiles.

Lipoprotein profiles were determined as described (35). Briefly, serum lipoprotein levels were analyzed by running 100 μL pooled serum onto a gel-filtration FPLC system. Samples were loaded on a GE Superose 6 10/30 GL column in 0.15 M sodium chloride containing 1 mM EDTA and 0.02% sodium azide, pH 7.4. Fractions (0.5 mL) were collected (0.5 mL/min). A lipid extraction kit (Biovision) was used for hepatic lipid extraction. The levels of total cholesterol and triglycerides in each fraction were determined by using cholesterol and triglycerides assay kits (SEKISUI Diagnostics).

### Human serum samples and measurement of circulating miR-483.

A group of 179 individuals were enrolled from the First Affiliated Hospital of Xi’an Jiaotong University during 2018–2019. The baseline characteristics of human subjects are summarized in Supplemental Tables 3 and 4. An amount of 3 mL whole blood was collected by venipuncture from median cubital vein after overnight fasting. After centrifugation at 1500*g* for 10 minutes, the serum was aliquoted into separator tubes, quickly frozen in liquid nitrogen, and stored at –80°C until use. The lipid profiles were detected by automatic chemical analysis (Hitachi LABOSPECT 008AS).

### Statistics.

All results are presented as mean ± SEM. Initially, data were tested for normality and equal variance to confirm the appropriateness of parametric tests. Experiments with 2 groups were compared by 2-tailed Student’s *t* test for parametric data or Mann-Whitney *U* test for nonparametric data. Experiments with more than 2 groups were compared by 1-way ANOVA with a Bonferroni’s post hoc test for parametric data or Kruskal-Wallis test with Dunn’s multiple comparisons for nonparametric data. All statistical analyses were performed with GraphPad Prism version 5.01; 2-tailed *P* values of less than 0.05 were considered statistically significant.

### Study approval.

The UCSD animal care personnel maintained all animals in accordance with NIH guidelines, and the IACUC of UCSD approved all experimental procedures (approval no. S12263). UCSD has an Animal Welfare Assurance document (A3033-01) on file with the Office of Laboratory Animal Welfare and is fully accredited by the Association for Assessment and Accreditation of Laboratory Animal Care (AAALAC) International. The study protocol for humans was approved by the ethics committees of Xi’an Jiaotong University. Written informed consent was obtained from all subjects.

## Author contributions

JYJS, MH, and JD conceived the original idea and designed the overall experimental plan. JD, MH, J. Li, AP, CW, JZ, YS, WTW, YZ, J. Liu, SCW, and PHH performed experiments. JD and MH interpreted the data and performed statistical analysis. ST, ZYY, and PLSMG provided essential input to the overall research plan. MH, JD, JYJS, ST, and PLSMG wrote the manuscript.

## Supplementary Material

supplemental data

## Version 1 10/29/2020

In-Press Preview

## Version 2 12/03/2020

Electronic publication
