# Supplementary material for: microRNA-483 ameliorates hypercholesterolemia by inhibiting PCSK9 production
Source: JCI Insight. 2020 Dec 3;5(23):e143812. doi: 10.1172/jci.insight.143812 (PMC7714402; doi:10.1172/jci.insight.143812)
Supplement: supplemental data [file jciinsight-5-143812-s047.pdf]

Supplemental file for

MicroRNA-483 Ameliorates Hypercholesterolemia by Inhibiting PCSK9 Production

Supplemental Figures 1-9
Supplemental Tables 1-4

Supplemental Figure 1

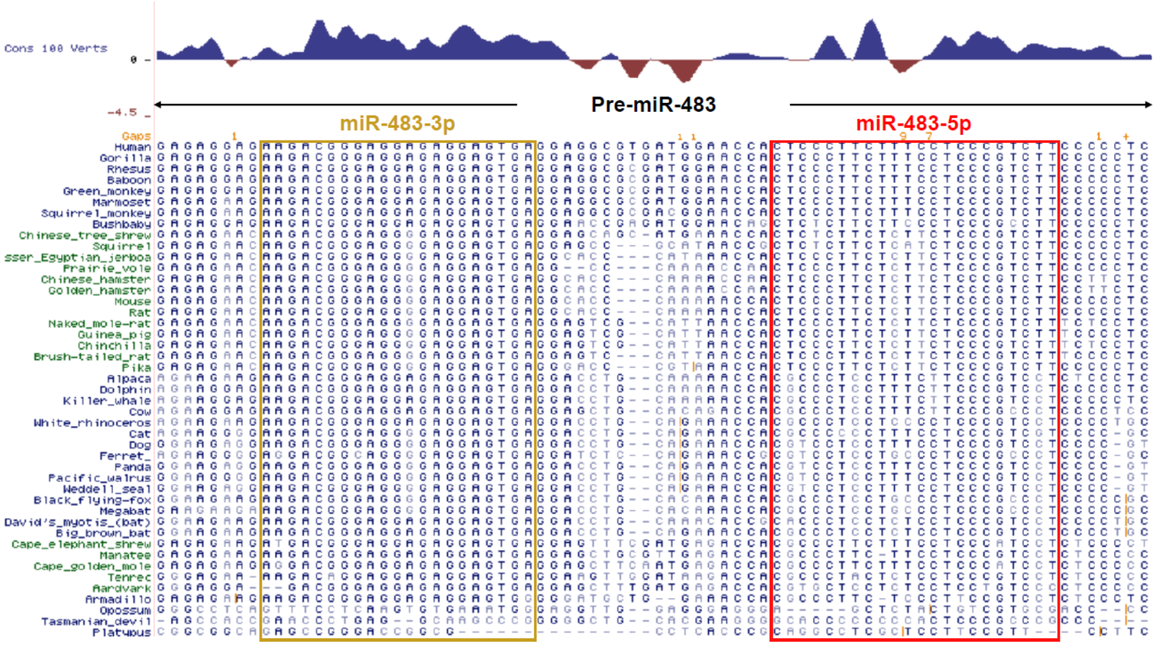

Supplemental Figure 1. The sequences alignment of miR-483-3p/-5p from multiple mammalian species.

Supplemental Figure 2

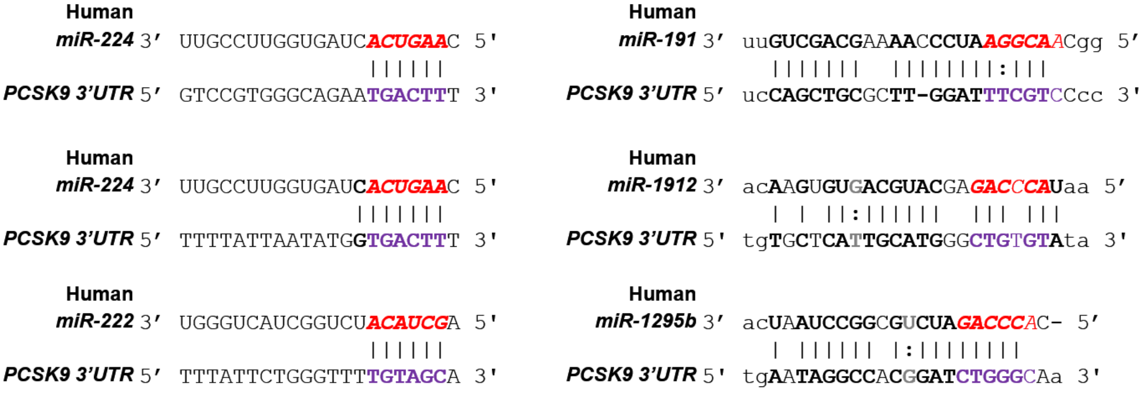

Supplemental Figure 2. Hsa-miR-224, -222, -191, -1912, and -1295b are predicted to target the 3'UTR of human *PCSK9*.

Supplemental Figure 3

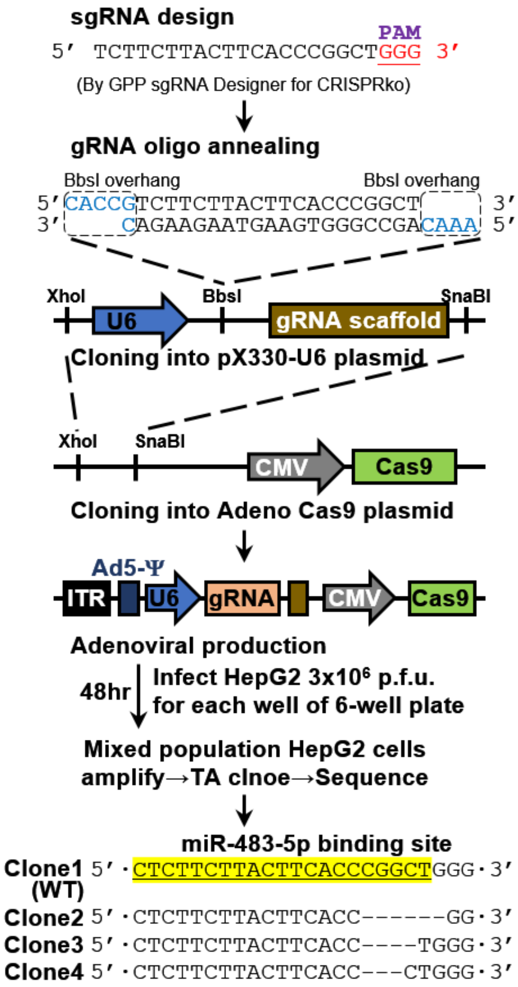

Supplemental Figure 3. CRISPR-Cas9 strategy for targeting the miR-483-5p binding site in human *PCSK9* 3'UTR. Single-guide RNAs (sgRNAs) were designed by using an online tool (https://portals.broadinstitute.org/gpp/public/analysis-tools/sgrna-design). Oligonucleotide pairs with BbsI-compatible overhangs were annealed and cloned into the vector pX330-U6-Chimeric_BB-CBh-hSpCas9 (Addgene). The XhoI restriction enzyme site was then introduced at the 5' end of the inserted gRNA and sub-cloned with SnaBI in the 3' end. The product including the gRNA and gRNA scaffold was inserted into the Adeno Cas9 plasmid (Addgene) for virus production. Adenovirus and empty control virus were enveloped by the UCSD Vector Development Core. HepG2 cells were infected with the adenovirus or empty control virus at 3 x 10^6 plaque-forming units to each well of a 6-well plate for 2 days. The mixed population of infected HepG2 cells was then selected for genotyping and downstream experiments.

Supplemental Figure 4

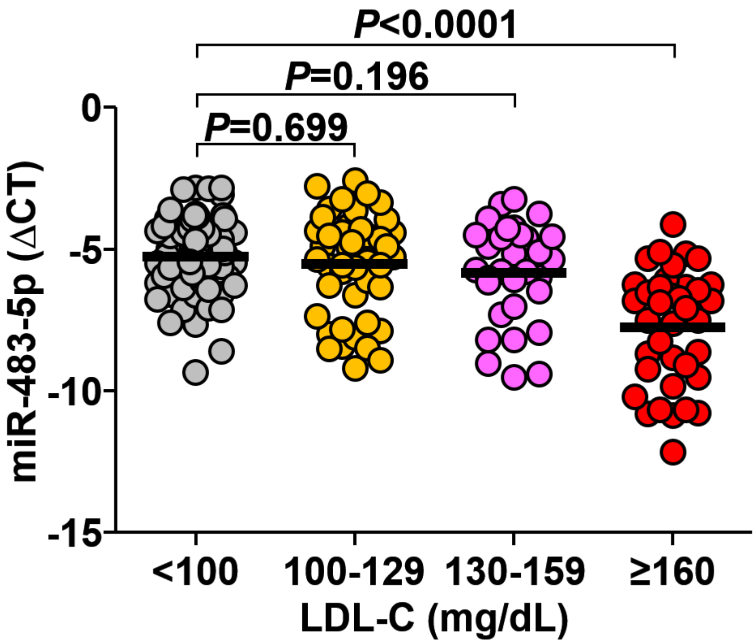

Supplemental Figure 4. Human subjects (n=179) were divided into 4 groups based on LDL-C levels: < 100 mg/dL (optimal, n=65, gray dots); 100-129 mg/dL (near/above optimal, n=46, orange dots); 130-159 mg/dL (borderline high, n=31, pink dots); ⩾ 160 mg/dL (high, n=37, red dots). Serum level of miR-483-5p was measured by qPCR with Cel-miR-39 as a spike-in control. ΔCT represents the difference between the cycle threshold of miR-483-5p and Cel-miR-39. *P* values were determined by Mann Whitney *U* test between 2 indicated groups.

Supplemental Figure 5

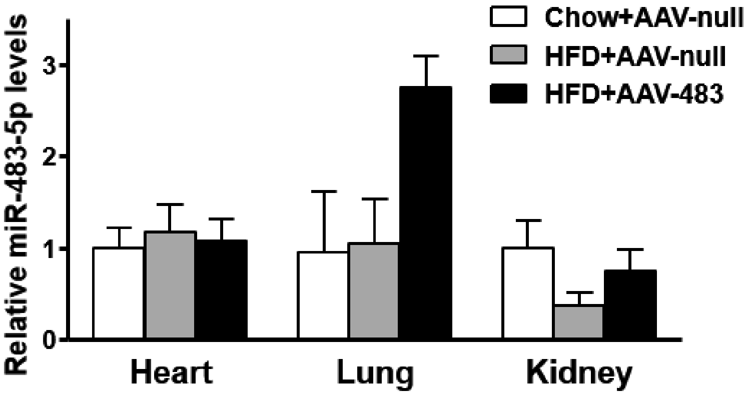

Supplemental Figure 5. C57BL/6 mice were fed chow or a high-fat diet (HFD) for 6 weeks. A single dose of AAV8-null (AAV-null) or AAV8-pri-miR-483 (AAV-483) ($1x10^{12}$ vector genome) was administered by tail-vein injection at the end of week 2. MiR-483-5p level in heart, lung and kidney tissue was quantified by qPCR. Data are mean ± SEM from 4-8 mice in each group.

Supplemental Figure 6

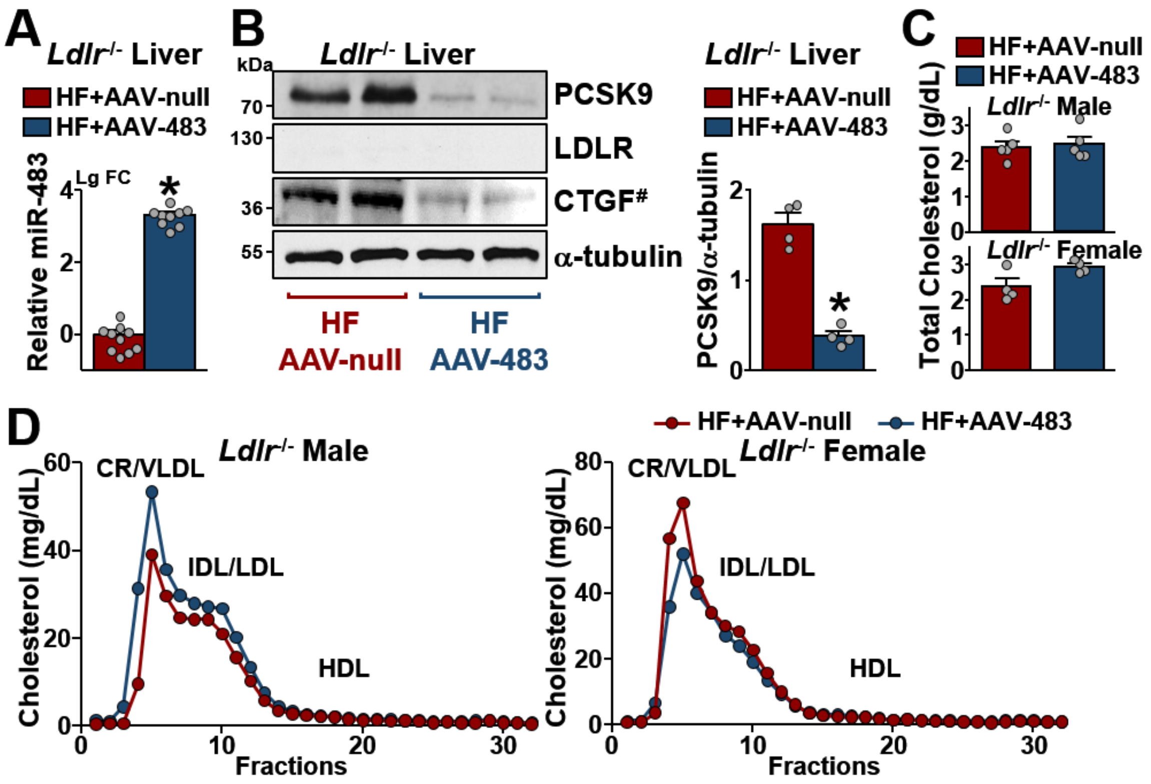

Supplemental Figure 6. (**A**-**D**) Male and female *Ldlr*-knockout mice were administered AAV-null or AAV-483 and fed an HFD for 6 weeks. Hepatic miR-483-5p levels were determined by qPCR, protein levels of PCSK9, LDLR, and CTGF were detected by western blot, # CTGF in the same samples were detected in parallel in a separate gel (A and B). Serum levels of total cholesterol, VLDL, LDL, and HDL (C and D) were determined by FPLC. The numbers of mice used are shown in Supplemental Table 1. Data are mean ± SEM. In (A), normally distributed data was analyzed by 2-tailed Student *t* test with Welch correction. In (B and C), non-normally distributed data were analyzed using Mann-Whitney *U* test. *P<0.05. IDL, intermediate-density lipoprotein.

Supplemental Figure 7

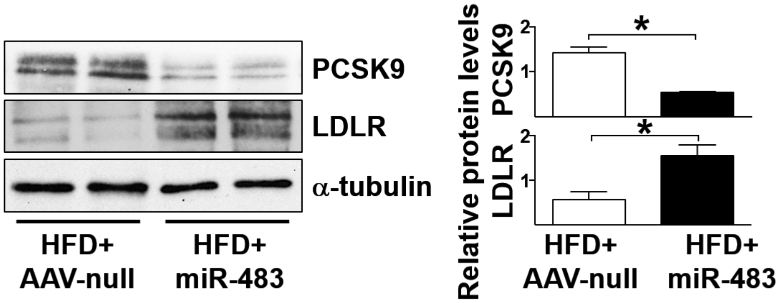

Supplemental Figure 7. C57BL/6 mice were fed an HFD for 6 weeks. A single dose of AAV8-null (AAV-null) ($4x10^{10}$ vector genome) or a low dose of AAV8-pri-miR-483 (miR-483) ($4x10^{10}$ vector genome) was administered by tail-vein injection at the end of week 2. Protein levels of hepatic PCSK9 and LDLR were detected by western blot analysis. Data are mean ± SEM from 4 mice in each group. **P*<0.05 with Mann Whitney *U* test between 2 indicated groups.

Supplemental Figure 8

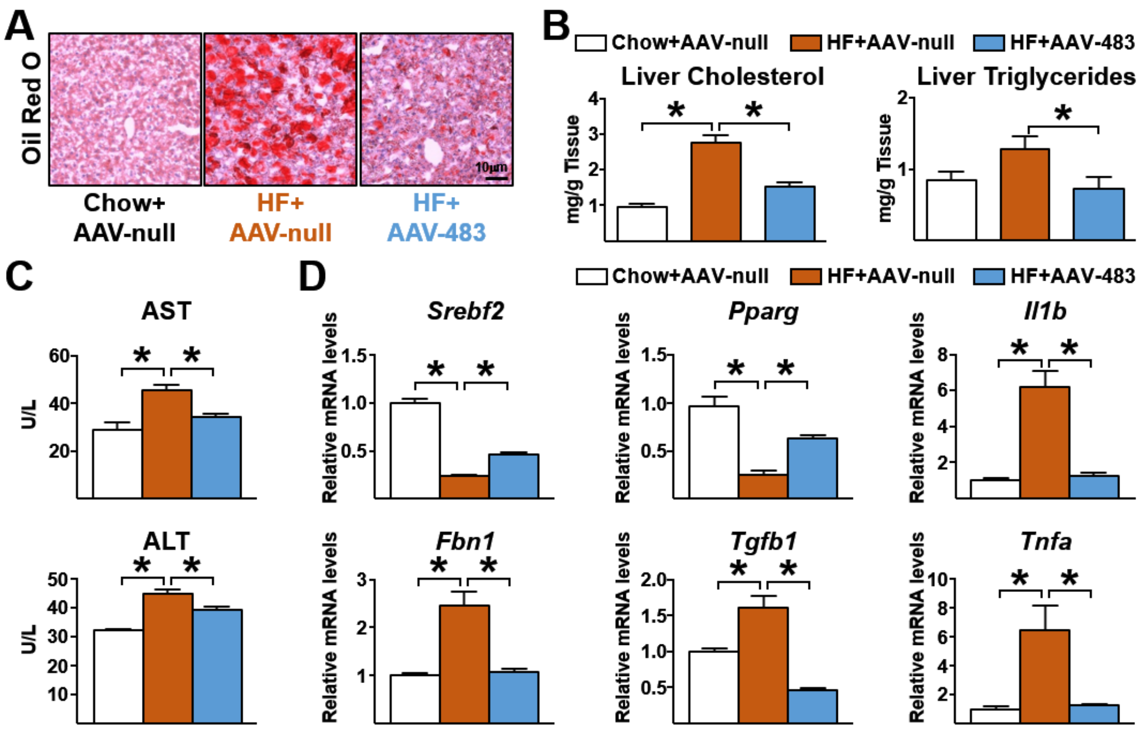

Supplemental Figure 8. Male and female C57BL/6 mice were fed chow or an HFD for 6 weeks. A single dose of AAV-null or AAV-483 was administered by tail-vein injection at the end of week 2. (**A**) Representative images show Oil-red O staining of liver sections. (**B**) Hepatic total cholesterol and triglycerides levels were measured by colorimetric assay. (**C**) Serum levels of aspartate transaminase (AST) and alanine transaminase (ALT) were detected by the kinetic method. (**D**) Hepatic mRNA levels of indicated genes were quantified by qPCR. Data are mean ± SEM (7-12 mice per group). In (B and C), normally distributed data were analyzed by 1-way ANOVA test with a Bonferroni *post-hoc* test between two indicated groups. Non-normally distributed data (D) were analyzed using Mann-Whitney *U* test between two indicated groups. *$P<0.05$.

Supplemental Figure 9

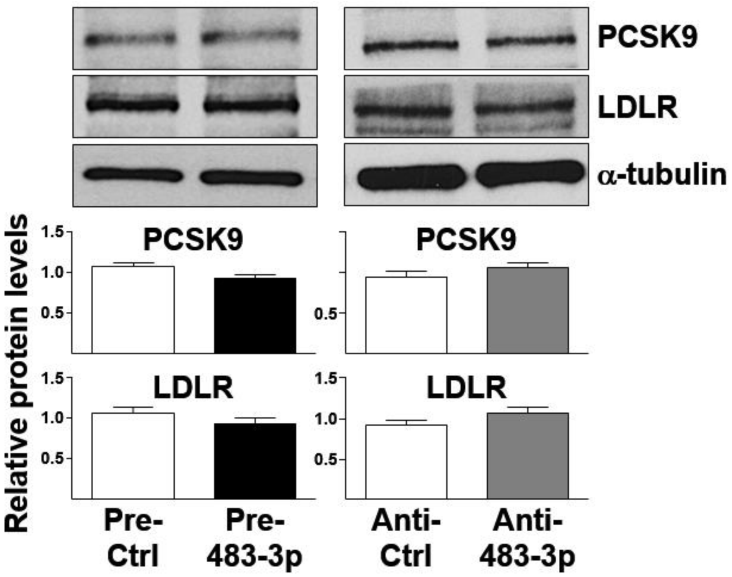

Supplemental Figure 9. HepG2 cells were transfected with pre-miR-483-3p mimic (pre-483-3p) or anti-483-3p for 24 hr. Levels of PCSK9 and LDLR protein were detected by western blot analysis. Data are mean ± SEM from 4 independent experiments, with comparisons to control groups arbitrarily set to 1.

Supplemental Table 1: Serum total cholesterol levels in experimental mouse groups

Total cholesterol (mg/dL)	Male (number)	Female (number)
C57Bl/6		
**Chow + AAV-null**	131.0 ± 4.340 (n=7)	93.04 ± 8.222 (n=7)
**HFD + AAV-null**	192.9 ± 4.280 (n=8)	138.2 ± 10.36 (n=7)
**HFD + AAV-483**	150.5 ± 5.240 (n=8)	99.28 ± 9.203 (n=6)
LDLR$^{-/-}$		
**HFD + AAV-null**	2379 ± 158.5 (n=5)	2361 ± 216.1 (n=4)
**HFD + AAV-483**	2469 ± 193.4 (n=5)	2922 ± 85.43 (n=4)
C57Bl/6		
**Ctrl**	191.0 ± 6.270 (n=3)	178.4 ± 11.30 (n=3)
**WT**	780.4 ± 48.26 (n=3)	372.7 ± 28.55 (n=3)
**WT+483**	166.7 ± 8.621 (n=4)	143.2 ± 9.610 (n=4)
**ΔBS**	514.1 ± 40.98 (n=7)	391.9 ± 58.06 (n=5)
**ΔBS+483**	451.1 ± 22.04 (n=6)	320.2 ± 11.83 (n=7)

AAV, adeno-associated virus; HFD, high-fat diet; Ctrl, control; WT, wild type

Supplemental Table 2: Primers used for RT-qPCR

Genes	Species	Sequence
ACTB	Homo sapiens	Forward: CATGTACGTTGCTATCCAGGC<br>Reverse: CTCCTTAATGTCACGCACGAT
Actb	Mus musculus	Forward: GGCTGTATTCCCCTCCATCG<br>Reverse: CCAGTTGGTAACAATGCCATGT
PCSK9	Homo sapiens	Forward: ATGGTCACCGACTTCGAGAAT<br>Reverse: GTGCCATGACTGTCACACTTG
Pcsk9	Mus musculus	Forward: TCTATGCTTCCTGCTGCCAT<br>Reverse: AGAGAGCCATGCAGGCATAT
LDLR	Homo sapiens	Forward: TCACCAAGCTCTGGGCGACG<br>Reverse: GTAGCCGTCCTGGTTGTGGCA
Ldlr	Mus musculus	Forward: TCAGACGAACAAGGCTGTCC<br>Reverse: CCATCTAGGCAATCTCGGTCTC
CTGF	Homo sapiens	Forward: AAAAGTGCATCCGTACTCCCA<br>Reverse: CCGTCGGTACATACTCCACAG
Ctgf	Mus musculus	Forward: GGCCTCTTCTGCGATTTCG<br>Reverse: GCAGCTTGACCCTTCTCGG
Srebf2	Mus musculus	Forward: GCAGCAACGGGACCATTCT<br>Reverse: CCCCATGACTAAGTCCTTCAACT
Tgfb1	Mus musculus	Forward: CCACCTGCAAGACCATCGAC<br>Reverse: CTGGCGAGCCTTAGTTTGGAC
Il1b	Mus musculus	Forward: ATGAGAGCATCCAGCTTCAA<br>Reverse: TGAAGGAAAAGAAGGTGCTC
Tnf1	Mus musculus	Forward: CCCTCACACTCAGATCATCTTCT<br>Reverse: GCTACGACGTGGGCTACAG
Fbn1	Mus musculus	Forward: TGTGGGGATGGATTCTGC<br>Reverse: AGTGCCGATGTACCCTTT
Pparg	Mus musculus	Forward: GGAAGACCACTCGCATTC<br>Reverse: GTAATCAGCAACCATTGG

Supplemental Table 3: Characteristics of human subjects

Parameters	n=179
**Male, n (%)**	91 (51)
**Age, years, median (range)**	47 (23-86)
**Body mass index, kg/m^2, median (IQR)**	24.09 (21.99-26.81)
**Waist, cm, median (IQR)**	85 (77-93)
**SBP, mmHg, median (IQR)**	115 (106-127)
**DBP, mmHg, median (IQR)**	77 (70-84)
**Laboratory data, median (IQR)**	
**Blood glucose, mg/dL**	81.7 (76.3-89.1)
**Triglycerides, mg/dL**	106.3 (78.8-158.6)
**Total cholesterol, mg/dL**	192.6 (156.6-233.6)
**LDL-C, mg/dL**	112.1 (87.4-154.3)
**HDL-C, mg/dL**	50.7 (43.7-58.4)

IQR: interquartile range, SBP: systolic blood pressure, DBP: diastolic blood pressure, LDL-C: low-density lipoprotein cholesterol, HDL-C: high-density lipoprotein cholesterol

Supplemental Table 4: Characteristics of humans with different LDL-C levels

	LCL-C level			
	**< 100 mg/dL**	**100-129 mg/dL**	**130-159 mg/dL**	**⩾ 160 mg/dL**
	n=65	n=46	n=31	n=37
**Male, n (%)**	37 (57)	20 (43)	19 (61)	15 (41)
**Age, years, median (range)**	40 (23-81)	52.5 (24-86)	54 (26-70)	49 (31-72)
**Body mass index, kg/m^2, median (IQR)**	23.62 (21.76-27.08)	24.10 (21.88-26.93)	24.09 (21.11-26.44)	24.34 (22.23-26.99)
**Waist, cm, median (IQR)**	85 (75-91)	84.5 (79-97)	86 (76-95)	85 (78-92)
**SBP, mmHg, median (IQR)**	115 (106-132)	115 (108-127)	118 (102-129)	114 (107-127)
**DBP, mmHg, median (IQR)**	79 (70-86)	77 (71-86)	75 (69-82)	76 (69-85)
**Laboratory data, median (IQR)**				
**Blood glucose, mg/dL**	80.1 (73.6-84.8)	81.6 (76.9-88.6)	85.1 (78.3-95.9)	83.5 (79.6-92.5)
**Triglycerides, mg/dL**	80.6 (62.0-103.8)	99.7 (83.3-124.0)	153.2 (111.6-197.5)	159.4 (121.4-200.2)
**Total cholesterol, mg/dL**	151.6 (138.1-162.8)	183.3 (170.9-199.5)	221.6 (215.4-228.2)	244.8 (237.1-263.0)
**LDL-C, mg/dL**	84.7 (77.7-88.9)	111.2 (107.1-116.4)	148.5 (141.1-154.3)	169.8 (166.3-179.8)
**HDL-C, mg/dL**	48.0 (39.4-56.5)	50.1 (43.7-58.8)	52.6 (44.1-62.3)	51.4 (1.21-64.6)
